# Recalcitrant Hailey-Hailey disease successfully treated with topical ruxolitinib cream and dupilumab

**DOI:** 10.1016/j.jdcr.2023.10.004

**Published:** 2023-10-21

**Authors:** Juna Khang, J. Michael Yardman-Frank, Li-Chi Chen, Hye Jin Chung

**Affiliations:** a Harvard Medical School, Boston, Massachusetts; b Harvard Combined Dermatology Residency, Boston, Massachusetts; c Department of Dermatology, Cutaneous Biology Research Center, Massachusetts General Hospital, Boston, Massachusetts; d Department of Dermatology, Beth Israel Deaconess Medical Center, Boston, Massachusetts; e Department of Dermatology, Harvard Medical School, Boston, Massachusetts

## Introduction

Hailey-Hailey disease (HHD) is a rare, chronic autosomal dominant dermatosis characterized by erythematous, painful vesicles and erosions at sites of friction. Individuals with HHD have a mutation on chromosome 3q 21-24 in the *ATP2C1* gene causing a defect in the Ca2+/Mn2+ ATPase secretory pathway in the Golgi complex, resulting in abnormal intracellular calcium homeostasis.^1^ This pump defect leads to impaired keratinocyte adhesion in the suprabasal layer of the epidermis.

There is currently no curative treatment for HHD. Management options aim to control symptoms and provide temporary relief. Therefore, there continues to be an unmet need for effective treatment options for this debilitating disease. We present the below case report as a novel treatment approach for HHD.

## Case report

**Fig 1:**
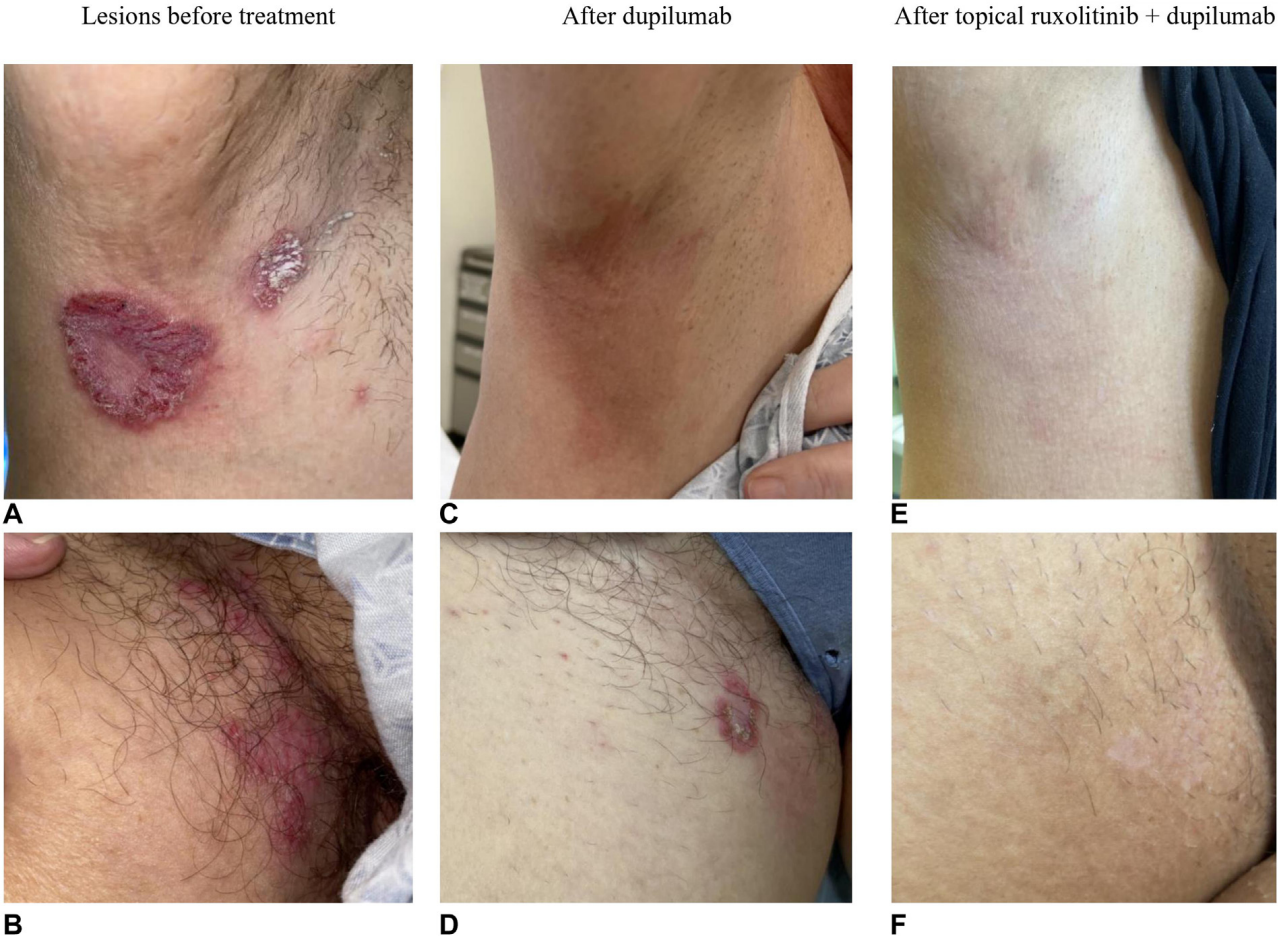
Lesions in the right axilla and inguinal fold (**A, B**) at baseline, (**C, D**) after dupilumab, and (**E, F**) after additional topical ruxolitinib cream.

This case is of a 53-year-old woman with a 25-year history of biopsy-proven HHD. She has experienced recurrent flares of painful erythematous thick plaques with fissuring and crusted vesicles/erosions in the axillae and inguinal folds, severely impacting her day-to-day life (Fig 1, *A* and *B*). She experienced minimal to no improvement with topical tacrolimus, oral antibiotics, topical erythromycin, and intralesional and topical steroids. She could not tolerate oral apremilast due to gastrointestinal side effects. The patient was started on dupilumab (300-mg/2-mL subcutaneous pen injector every 14 days). After 2 weeks, she reported marked improvement of HHD on her axillae, but the lesions on her inguinal folds persisted, causing significant pain (Fig 1, *C* and *D*). Topical ruxolitinib 1.5% cream twice a day was subsequently initiated. She experienced marked improvement of residual lesions within 1 day and complete resolution by 1 month (Fig 1, *E* and *F*). During a 5-month follow-up, the patient did not note any side effects from the ruxolitinib and dupilumab treatment or flare-ups of her HHD.

## Discussion

To our knowledge, this is the first case report of recalcitrant HHD successfully treated with a topical Janus kinase (JAK) inhibitor as well as dupilumab. JAK inhibitors are a new class of targeted therapeutics that inhibit the JAK-STAT signaling pathway, which involves numerous cytokines, such as interferon alfa/beta, interferon gamma, interleukin (IL) 2, IL-4, IL-5, IL-6, IL-12, IL-13, IL-15, and IL-23.^2^ One prior case report has shown improvement in treatment-refractory HHD with the oral JAK1 inhibitor abrocitinib.^3^ As our patient had multiple comorbidities and her HHD was limited to the inguinal folds after dupilumab therapy, a topical rather than oral JAK inhibitor was used. Notably, our patient reported rapid improvement of her lesions within 1 day without significant irritation. The basis of HHD pathogenesis is loss of cellular adhesion due to haploinsufficiency of ATP2C1, and these defects create cellular stress, promoting apoptosis of keratinocytes and release of inflammatory cytokines.^1^ Thus, the benefit of topical ruxolitinib seen in this patient may be due to the blockade of these cytokine-driven pathways and decreasing inflammatory cell infiltration.

Few prior case reports have illustrated treatment of refractory HHD with dupilumab.^4^ In HHD, there appears to be defective actin polymerization as well as impaired adherens protein assembly.^5^ In normal keratinocytes, increased extracellular Ca+ leads to actin reorganization and decreased intracellular adenosine triphosphate, a process hindered in HHD.^5^ Previous cellular models have illustrated that IL-4 stimulates eotaxin-3 agonizing CCR3 (which attracts eosinophils, basophils, and Th2 lymphocytes) and antagonizes CCR1 and CCR5 (which inhibits increases in intracellular calcium and actin depolarization).^5^^,^^6^ As such, dupilumab may benefit patients with HHD by both decreasing inflammatory infiltrative cells and stopping further hindrance of actin depolarization.

Ultimately, our case report adds to the nascent literature on the utility of dupilumab and topical JAK inhibitors for refractory HHD. Although the exact mechanisms of their action in HHD are unclear, our novel combination of topical ruxolitinib and dupilumab may work through decreasing inflammatory infiltration and potentially promoting cellular adhesion, offering a relatively safe combination therapy for a challenging disease.

## Conflicts of interest

None disclosed.
